# Environmental Symbiont Acquisition May Not Be the Solution to Warming Seas for Reef-Building Corals

**DOI:** 10.1371/journal.pone.0013258

**Published:** 2010-10-07

**Authors:** Mary Alice Coffroth, Daniel M. Poland, Eleni L. Petrou, Daniel A. Brazeau, Jennie C. Holmberg

**Affiliations:** 1 Graduate Program in Evolution, Ecology and Behavior, Department of Geology, University at Buffalo, Buffalo, New York, United States of America; 2 Department of Biological Sciences, University at Buffalo, Buffalo, New York, United States of America; 3 Department of Pharmaceutical Sciences, University at Buffalo, Buffalo, New York, United States of America; Monash University, Australia

## Abstract

**Background:**

Coral reefs worldwide are in decline. Much of the mortality can be attributed to coral bleaching (loss of the coral's intracellular photosynthetic algal symbiont) associated with global warming. How corals will respond to increasing oceanic temperatures has been an area of extensive study and debate. Recovery after a bleaching event is dependent on regaining symbionts, but the source of repopulating symbionts is poorly understood. Possibilities include recovery from the proliferation of endogenous symbionts or recovery by uptake of exogenous stress-tolerant symbionts.

**Methodology/Principal Findings:**

To test one of these possibilities, the ability of corals to acquire exogenous symbionts, bleached colonies of *Porites divaricata* were exposed to symbiont types not normally found within this coral and symbiont acquisition was monitored. After three weeks exposure to exogenous symbionts, these novel symbionts were detected in some of the recovering corals, providing the first experimental evidence that scleractinian corals are capable of temporarily acquiring symbionts from the water column after bleaching. However, the acquisition was transient, indicating that the new symbioses were unstable. Only those symbiont types present before bleaching were stable upon recovery, demonstrating that recovery was from the resident *in situ* symbiont populations.

**Conclusions/Significance:**

These findings suggest that some corals do not have the ability to adjust to climate warming by acquiring and maintaining exogenous, more stress-tolerant symbionts. This has serious ramifications for the success of coral reefs and surrounding ecosystems and suggests that unless actions are taken to reverse it, climate change will lead to decreases in biodiversity and a loss of coral reefs.

## Introduction

Modern coral reef ecosystems are based on and maintained by the symbiosis between corals (Cnidaria: Hexacorallia: Scleractinia) and photosynthetic dinoflagellate symbionts (Alveolata: Dinophycea: *Symbiodinium*). Rising sea surface temperatures (SSTs) [1] threaten this ecologically important symbiosis [2]–[5] as SSTs only slightly above the annual mean can result in a loss of the algal symbionts from the host, a phenomenon termed ‘bleaching’ [6]–[7]. Loss of symbionts deprives the coral of a major source of nutrients and severe bleaching can lead to coral death with significant ramifications for the reef ecosystem.

Scleractinian corals vary in their susceptibility to bleaching and this may be a reflection of the symbiont type within the coral, as symbiont taxa exhibit different tolerances to stress [6]–[11]. It has been proposed that reef corals might recover from and adapt to bleaching events by acquiring more stress-tolerant symbionts from the surrounding environment [12]. However, evidence for changes in a colony's endosymbionts is lacking. The majority of corals initially obtain symbionts from the surrounding environment at the larval or single polyp stage [13]. Although multiple symbiont types are initially acquired, selectivity exists, as not all available symbiont strains are taken up and only a subset of those strains are retained [14], [15].

For an adult coral to survive and subsequently recover after a bleaching event, the coral must either retain symbionts that can meet its minimum physiological requirements or acquire the necessary symbionts from the environment after bleaching. Some corals may naturally contain stress-tolerant symbionts that dominate the symbiosis, in which case bleaching should be minimal, and recovery rapid [6], [7], [16]. In fact, some corals recover from bleaching by repopulation from background stress-tolerant *in hospite* symbionts remaining within the host after the bleaching-induced stress (i.e., those that are usually present at low to undetectable levels within the host prior to bleaching) [7], [10]. On the other hand, if corals lack these stress-tolerant symbionts, then post-bleaching recovery depends on the acquisition of a more stress-tolerant symbiont from the surrounding environment. While anemones and octocorals are able to acquire *Symbiodinium* from exogenous sources (i.e. the environment) [17], [18], this ability has not been demonstrated for the cnidarians that provide the structural foundation of the coral reef ecosystem, the scleractinian corals. Using the Caribbean finger coral *Porites divaricata*, we show that although scleractinian corals may have the ability to acquire exogenous *Symbiodinium*, the new associations were unstable.

## Materials and Methods

### Study Organism and Field Surveys

This work examined symbiont change within *Porites divaricata*, a common shallow water scleractinian found throughout the Caribbean and was conducted in the Florida Keys National Marine Sanctuary (FKNMS) under permit FKNMS-2005-008 to M.A. Coffroth. To first establish the diversity of *Symbiodinium* within *P. divaricata*, colonies were sampled from six locations on the bay and ocean side of the upper, middle and lower Florida Keys (n = 18–54 per site, total n = 182). Symbiont strain within each colony was determined based on sequence variation within a 0.2 kb segment of Domain V of the chloroplast cp23S rDNA (cp-typing, [19]; see below).

### Infection Experiment

To test the ability of *P. divaricata* to acquire exogenous symbionts, we exposed experimentally bleached corals to the novel *Symbiodinium* types A188, B211, B224 and D206. This nomenclature is based on phylogenetic clade [i.e., A] and length [i.e., 188 bp] of a variable region in the chloroplast 23S rDNA gene [19]. The symbiont types used for the infection experiment differed from the symbiont type that typically dominates in these corals (*Symbiodinium* B170). For these experimental manipulations, *P. divaricata* colonies (n = 105) were collected from one of our survey sites, the middle keys, ocean side (N24° 49.791′ W80° 45.743′). *Symbiodinium* types and symbiont densities within these colonies were determined prior to experimental manipulations. After sampling for the pre-treatment symbiont assemblage, the corals were placed into two 75.6 liter glass aquaria that contained 20 L of 1 µm filtered seawater (FSW) and treated with antibiotics [20] for 24 h to deter bacterial infections. Water within the tanks was aerated and recirculated at a high flow rate and the aquaria were placed under plant grow lights with a 14 h:10 h light:dark cycle. Light measurements, recorded by Hobo temperature and light loggers (Onset Computer Corp., Bourne, MA USA) within in each aquarium, were measured in lux. Although these measurements cannot be converted to the more traditional measurement of PAR (µE), comparisons between laboratory and field values provide a relative measure of light levels. The mean illumination within our experimental system was 268 lux while *in situ* values ranged from 75.5 to 2513 lux with a mean 615 lux. Water temperature was maintained at 26°C (the average temperature *in situ*) and partial water changes were done once every three days. After a 4 d acclimatization period, 15 corals were placed in an aquarium with water temperature maintained at 26°C for the duration of the experiment which served as the “non-bleached” control group. The remaining coral colonies were randomly distributed among 4 aquaria and water temperature was slowly raised to 33°C over 14 d and then maintained at this temperature for an additional 13 d.

At 25 d the corals were again treated with antibiotics. After the 27 d at elevated temperature, all of the coral colonies were sampled again to determine the genotype and cell density of *Symbiodinium* within the host tissue. Subsequently, the bleached colonies were randomly divided into five treatment groups (n = 14–15) in aquaria with 20 L of circulating, aerated, and filtered sea water at ambient temperature. One group served as a negative control in which bleached corals (n = 15) were not inoculated with novel symbionts, i.e. the “bleached control”. This control is in addition to the non-bleached controls (n = 15) that were maintained at a constant temperature of 26°C throughout the study. The latter group, also not inoculated, served as a control for the other factors in the experimental set-up (i.e., containment, light levels, etc.). The remaining bleached *P. divaricata* were exposed to one of four *Symbiodinium* types A188, B211, B224 and D206 [19] (equivalent to D1a [21]) for four weeks in laboratory aquaria. Members of Clade D (including D1a/D206) have been proposed to be stress-tolerant [6]–[8], [22]–[24]. These four *Symbiodinium* types are found in the Florida Keys but were not seen in symbiosis with *P. divaricata* in our field surveys throughout the Florida Keys and thus served as a marker for the exogenous uptake of symbionts. The cultures had been reared in the laboratory following the methods described by [25] and were maintained at a concentration of 1,000 cells ml^−1^ in each aquarium. Each aquarium was inoculated with a novel symbiont strain for a month to mimic the continuous availability of symbionts in the field. Inoculations occurred once every three days after water changes, and ended five days before the last sampling period.

All aquaria were loosely covered with clear plastic to limit cross-contamination and the water temperature returned to 26°C during inoculation and recovery phase. During this period, the corals were sampled at three weeks and at five weeks after the heat stress had ended. Five days before the last sampling (5-wks), inoculations were terminated to ensure that no symbionts remained in the coral's gut. Prior to sampling, the corals were thoroughly rinsed in filtered seawater to remove any *Symbiodinium* that may have adhered to the coral surface. This treatment included two separate rinses with vigorous shaking of the branch in FSW and a final rinse in FSW prior to sampling. In addition, all the sampling equipment was rinsed in tap water after each sampling to reduce contamination.

### Symbiont Homogeneity throughout Colony

It was necessary to determine if individual *P. divaricata* colonies contain a single dominant symbiont throughout the colony to verify the accuracy of results obtained through resampling the same colony over the course of the experiment. In addition to the colonies used in the experimental manipulations, five other *Porites divaricata* colonies were collected from the same site in the middle keys. These five colonies were sampled in different positions on each of 3 branches (inner vs. outer, top vs. bottom) and symbiont type was determined as described below.

### Molecular Analysis

#### Extractions and symbiont identity

*Porites divaricata* tissue was preserved in salt-saturated DMSO and total DNA was later extracted and quantified following the protocol of [26]. Subsequently, a 0.2 Kb segment of Domain V of the chloroplast (cp23S)-rDNA was amplified using the polymerase chain reaction (PCR) with the primer pairs 23SHYPERUP and 23SHYPERDNM13 following the protocol of [19]. The resulting PCR products were run on a 6.5% Long Ranger (FMC Bioproducts, Rockland, ME)/1X Tris Borate (TBE) polyacrylamide gel and visualized on a LI-COR 4200 NEN® Global IR2 DNA sequencing system (LI-COR Biosciences, Lincoln, NE, USA) under the conditions described by [19]. This technique has a detection resolution of 10 to 1,000 cells [19]. The samples were subsequently compared with size standard ladders and cultures of known *Symbiodinium* to determine the identity of the cp 23S-rDNA fragments.

#### Quantitative PCR (qPCR) of types B224 and D206

A type-specific primer for *Symbiodinium* B224 was developed using the chloroplast 23S rDNA molecule from alignments of several closely related *Symbiodinium* clade B cultures [19] and checked using PRIMER3 ver. 0.4.0 (http://frodo.wi.mit.edu/). The forward primer amplifies all *Symbiodinium* (23SHYPERUP, [19]), but a type-specific reverse primer (5′-AAT GTT GGG TCG AAC AGA AAA -3′) allowed for B224-specific assays. Primers were screened against other cultured *Symbiodinium* A, B, C and D and resulted in type-specific amplicons for *Symbiodinium* B224 only. For clade D assays we used Universal FP and D-specific RP to amplify the nuclear SSU and ITS1 rDNA interface as described in [27].

Sample DNA concentrations were determined using a NanoDrop ND-1000 Spectrophotometer (NanoDrop Technologies, Wilmington, DE) and were normalized before reactions. Assays were performed on a Stratagene Mx3005P QPCR System (Stratagene, La Jolla, CA.). *Symbiodinium* B224 was amplified in 20 µL volumes containing 1X PCR Buffer, 200 µM of each dNTP, 2.0 units of *Taq* DNA polymerase, 500 nM of each primer, 0.5 µL each of SYBR Green I dye and ROX reference dye, and 5–10 ng of template DNA. The thermal conditions were 5 min at 94°C initial denaturing, followed by 35 cycles of 30 s at 94°C, 30 s at 50°C and 30 s at 72°C. Additional cycles of 1 min at 95°C, 30 s at 55°C, and 30 s at 95°C were performed to observe the dissociation (melting) curve of qPCR products. *Symbiodinium* Clade D specific assays were conducted in 20 µL volumes containing 1X PCR Buffer, 200 µM of each dNTP, 2.0 units of *Taq* DNA polymerase, 100 nM of each primer, 0.5 µL each of SYBR Green I dye and ROX reference dye, and 5–10 ng of template DNA. Thermal cycle conditions were 5 min at 94°C initial denaturing, followed by 40 cycles of 30 s at 94°C, 33 s at 64°C and 45 s at 72°C. Additional cycles, as described above for *Symbiodinium* B224 assays, were followed to observe the dissociation curve. Threshold cycle values (C_t_: the cycle at which a significant signal in fluorescence above background level occurs) below 28 were considered as positive results for B224-specific assays and below 34 for D-specific assays. In the B224-specific qPCR, the specificity is established by the B224-specific reverse primer that only anneals to *Symbiodinium* B224. However, the forward primer (23SHYPERUP) in this assay anneals to non-target *Symbiodinium* DNA, leading to the amplification of low amounts of non-target *Symbiodinium* DNA in the B224-specific qPCR assays. The non-target amplicons caused the C_t_ threshold to be shifted so that the C_t_ threshold was adjusted toward the lowest detection limit of the standards and those of non-target DNA controls. Melting curve analyses show the presence of a second peak (at approximately 86°C) that is offset from that expected of target DNA (82°C). Thus, the dissociation curve analyses always provided additional confirmation of the presence/absence of target DNA at 82°C. In competitive DNA trials with target DNA comprising 50% (5 ng DNA/µL), 10%, 1%, 0.1% and 0.01% (0.001 ng DNA/µL) mixed with non-target *Symbiodinium* DNA for a total of 10 ng DNA/µL per trial, we achieved a detection limit of 0.01% for clade D among other DNA, and 0.1% for *Symbiodinium* B224. Standard curves were generated with seven 10-fold serial dilutions of cloned clade D ITS-rDNA and *Symbiodinium* B224 23S-rDNA gene fragments. Each *P. divaricata* sample was run in duplicates and each assay included serial dilutions of cloned target DNA for standard curve along with non-template and positive controls. The efficiency of the qPCR assays were approximately 95±2% between runs for clade D and 117±2% for B224.

Because we have no information on the number of rDNA copies per *Symbiodinium* cell, we provide no information on the absolute or relative abundance of *Symbiodinium* in both assays. Here, we only report the presence or absence of specific *Symbiodinium* types that may not be detectable by traditional PCR methods.

All samples from the Clade D and B224 treatments were screened with the appropriate specific qPCR primers. To verify that Clade D was not a background symbiont in *P. divaricata*, an additional random subsample of the pre-bleached and post-bleached (pre-inoculation) samples (n = 50 each) were screened using the Clade D specific qPCR primers and all lacked *Symbiodinium* D.

### Symbiont enumeration

Symbiont density within coral tissues was determined three times over the course of the experiment; (1) before exposure to elevated temperature, (2) when the heat treatment was terminated and (3) 5 weeks into the recovery period. Coral tissue was scraped from the colony surface and placed in 1.0 ml of 5% formalin. The length and width of the scar were measured and these dimensions (length x width) were used to estimate the surface of tissue removed. Subsequently, each tissue sample was homogenized and 9 µL aliquots were counted using a hemacytometer. A total of four replicate counts were conducted per tissue sample and mean symbiont density per mm^2^ was calculated.

## Results

### Field surveys

**Figure 1:**
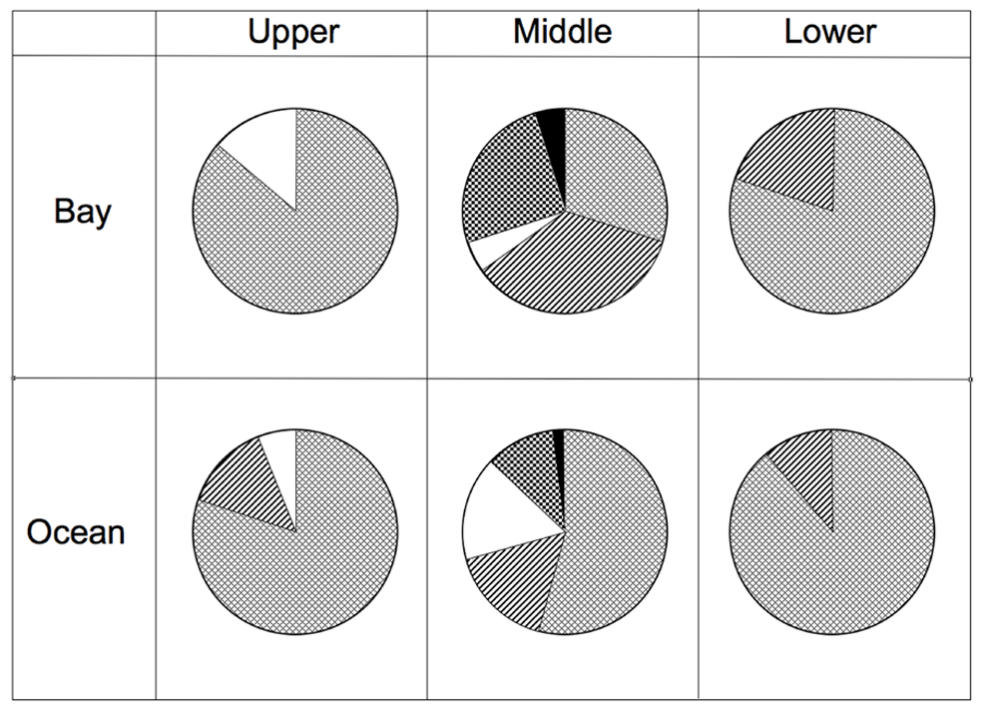
Percentage of *Porites divaricata* colonies that harbor a given *Symbiodinium* strain in field surveys. Colonies were collected from the bay and ocean side of the Upper (n = 30, 30), Middle (n = 20, 54) and Lower Keys (n = 30, 18, bay and ocean respectively) in 2004. Color code: Light hatching- B170; Diagonal- B170+A194; White – B170+ others; (others  = 160, 178, 190, 196 or 178+194)) Dark hatching – A194; Black – *Symbiodinium* 178 alone or with A194.

In an initial survey, *P. divaricata* collected from six sites in the Florida Keys harbored primarily *Symbiodinium* B170 (Fig. 1). Other symbiont types were found in lower abundance and included *Symbiodinium* Clade B184 and B178 and less frequently *Symbiodinium* Clade A194. The field surveys thus demonstrated the ability of *P. divaricata* to harbor multiple symbiont types.

### Symbiont Homogeneity Throughout Colony

Replicate sampling at multiple locations across five colonies collected from the ocean side of the middle key site verified that the dominant symbiont types did not vary with sampling location within individual colonies. All colonies harbored the dominant symbiont type B170 throughout the colony. These data confirmed that resampling a colony over the course of the experiment provided a representation of the symbionts present.

### Symbiont Densities

**Figure 2:**
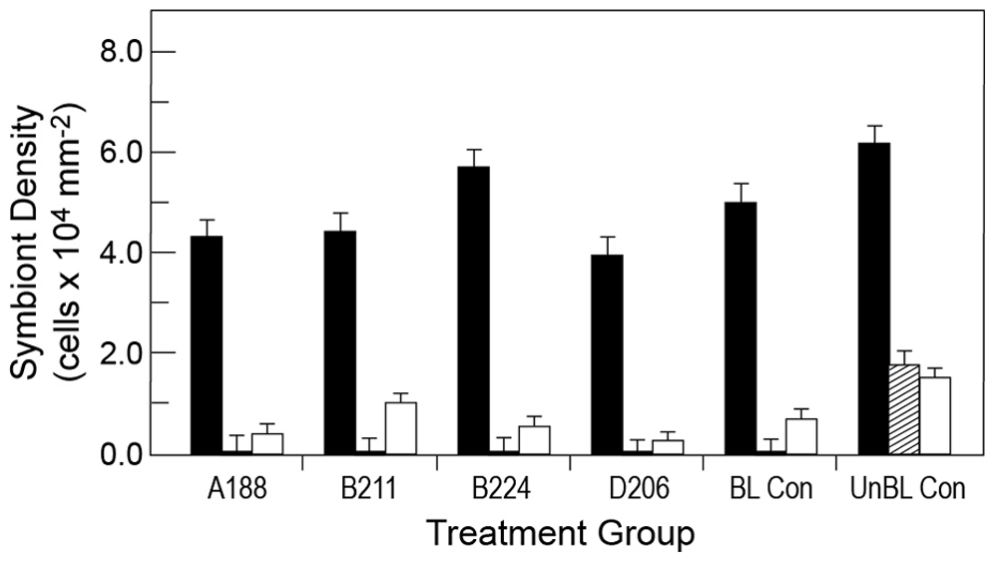
*Symbiodinium* densities within colonies of *P. divaricata*. Prior to heat treatment (black), after one month of elevated temperature (diagonal lines, center column) and after a recovery period at ambient temperatures for five weeks with exposure to exogenous *Symbiodinium* (white). Symbiont densities were enumerated using a hemocytometer. Treatment group indicates the type of *Symbiodinium* that was added to the tank during the recovery period (+5 wks) or the two control treatments where corals were (1) bleached but not inoculated with exogenous symbionts (BL Con) or (2) not induced to bleach nor inoculated with exogenous symbionts (UnBL Con). Error bars: Standard Deviation.

**Figure 3:**
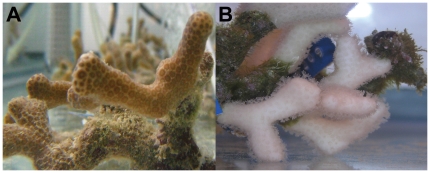
*Porites divaricata* colonies. (A) before, and (B) after one month at elevated temperature.

Prior to bleaching, cell counts ranged from 3.97 to 6.18×10^4^ cells cm^−2^ (Fig. 2). Heat-induced bleaching eliminated 98–99% of the original symbiont population (Fig. 2 and 3). The unbleached control colonies, maintained at ambient temperature, also experienced a decline in symbionts densities (approx 70%) (Fig. 2), suggesting that our study system was affected by other experimental stressors, such as reduced ambient light level or containment. Nonetheless, the heat treatment resulted in a significant reduction in symbiont densities compared to both pre-bleached levels and the non-bleached controls (repeated measures ANOVA using Greenhouse-Geisser correction for violation of assumption of sphericity, *F*(1.088,88.144) = 137.119, *p*<0.001; within-subject contrasts, *F*(1,81) = 153.654, *p*<0.001). Furthermore, despite other potential stressors, symbiont numbers in the colonies increased once thermal stress was discontinued (Fig. 2).

### Infection Experiment

#### cp-23S-rDNA Screening for Novel Symbiont Types

**Figure 4:**
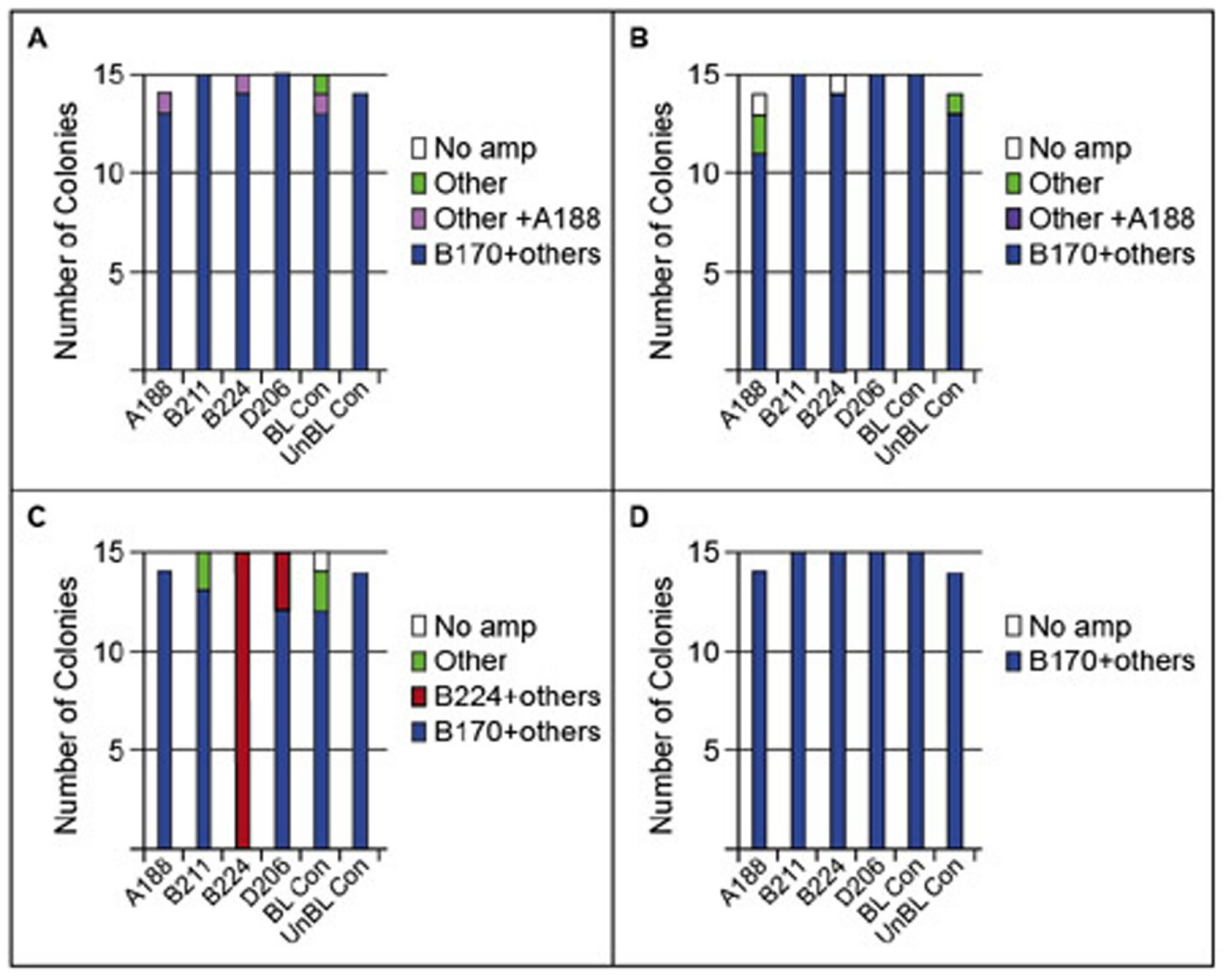
Number of colonies with a given *Symbiodinium* type in each treatment detected using cp-23S-rDNA. (**A–D**) Symbiont communities (A) prior to heat-induced bleaching (PreBL), (B) after corals had lost up to 90% of their symbionts at the end of heat treatment (PostBL), (C) after three and (D) five weeks of exposure to a novel symbiont type “Other”  =  types B178, B184 and/or A194 found alone and “B170+ other”  =  B170 found alone or in conjunction with types B178, B184 and/or A194. “Other + A188”  =  type A188 found at low levels with B170+ others in 3 colonies; “B224+other”  =  types B170, B178 and B184 found in conjunction with B224. Note that B224 was also detected at low levels in 3 of 15 colonies in the D206 treatment during the 3 wks sampling (C). B224 was not detected in any other treatment or in field surveys of over 180 colonies (Fig. 1). Given that the D206 tank was adjacent to the B224 treatment tank the B224 tank is the most likely source of the B224 contamination.

Colonies used in the infection experiment study were collected from the middle keys, oceanside site. Analysis of symbiont type within these corals showed that prior to bleaching, the symbiont strains within the experimental colonies resembled those found in the field surveys of *P. divaricata* (Fig. 1). *Symbiodinium* B170, the dominant symbiont type, was found alone in 82% and with other types in 17% of the colonies (Fig. 4A, prebleached samples). *Symbiodinium* A188, which hitherto had been undetected in *P. divaricata*, was observed at very low levels in 3.4% of the colonies in combination with the dominant symbiont, B170 (Fig 4A). Aside from A188, the novel *Symbiodinium* types used in the infection study (A188, B211, B224 and D206) were not detected in colonies prior to or immediately after bleaching using *Symbiodinium*-specific primers for the variable region within the chloroplast 23S-rDNA gene (cp-23S-rDNA), as well as quantitative PCR (qPCR) with B224- and Clade D-specific primers (Fig. 4A–B).

After heat-stress and prior to the addition of the novel symbionts, molecular analysis showed that the *Symbiodinium* types previously encountered in *P. divaricata* still dominated the symbiosis (i.e., 77% of the colonies contained only *Symbiodinium* B170 and 16% of the colonies contained a mixture of B170 and others, Fig. 4B, post bleached samples). However, within some individual colonies, *Symbiodinium* types not initially detected were later detected when the colonies underwent bleaching, suggesting the presence of viable, background types (Fig. 4B) remaining in the host after bleaching and whose presence had previously been masked by the dominant types. These background symbiont types (e.g., A194, B178, B184) had been detected in other colonies prior to bleaching (Fig. 4A).

After exposure to the novel symbiont types for three weeks, *Symbiodinium* B170 remained the predominant symbiont type (Fig. 4C). Cp-23S-rDNA screening did not detect the novel *Symbiodinium* types A188, B211 or D206 (Fig. 4C). In contrast, although B170 remained the dominant symbiont, the novel symbiont *Symbiodinium* B224 was detected in all 15 colonies from the B224 treatment after 3 wks, but was not detected using this technique after 5 wks (Fig. 4C and 4D). *Symbiodinium* B224 was also temporarily detected at low levels in 3 of 15 colonies in the D206 treatment during the 3 wks sampling (Fig. 4C). B224 was not detected in any other treatment or in field surveys of over 180 colonies (Fig. 1). Given that the D206 tank was adjacent to the B224 treatment tank, the B224 tank is the most likely source of the B224 contamination.

#### Quantitative PCR (qPCR) of types B224 and D206

**Figure 5:**
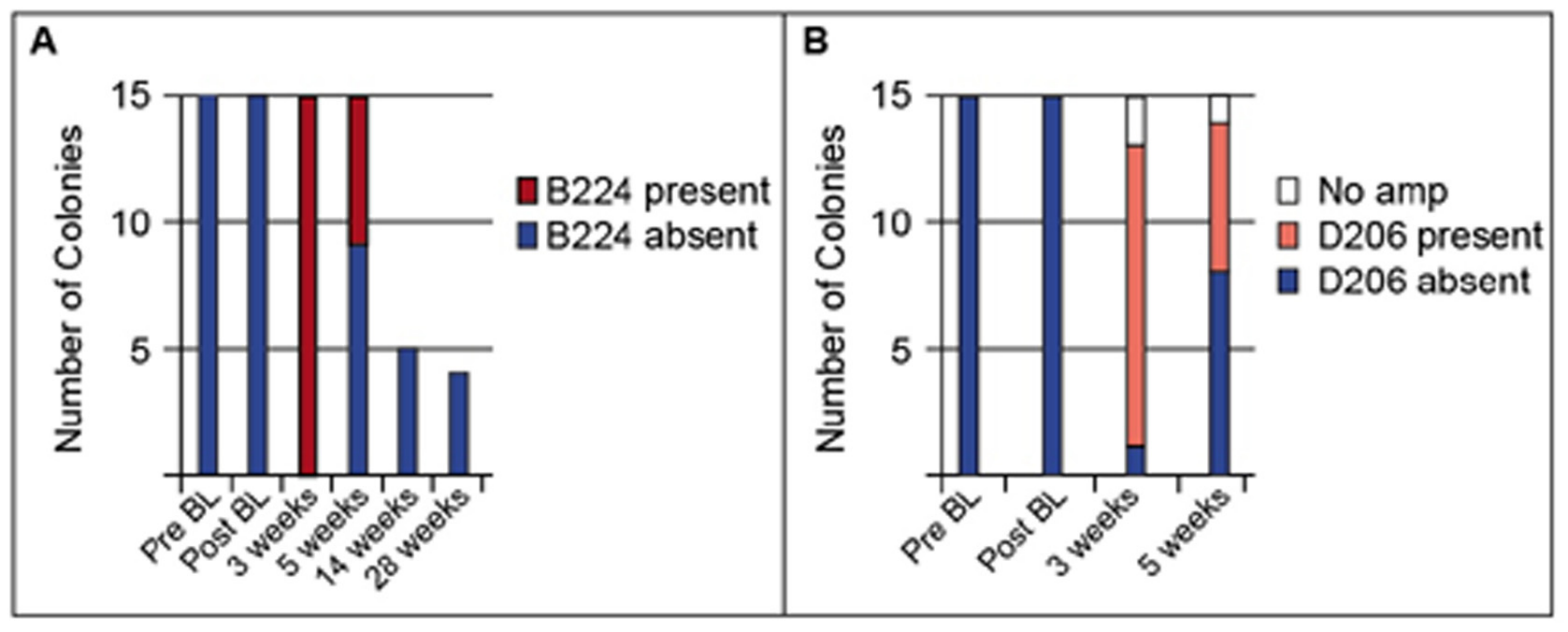
Number of colonies with a given *Symbiodinium* type in the B224 and D206 treatments detected using qPCR primers specific. qPCR primers were employed for secondary screening in the (A) B224 treatment and (B) D206 treatment. Sampling times- Pre BL; Post BL 3 wks and 5 wks as in Figure 2 (A–D); After 14 and 28 wks: colonies from the B224 treatment were returned to the field where lower sample sizes indicate loss of colonies from storms.

Using a more sensitive technique with type-specific qPCR primers, *Symbiodinium* B224 was found in all corals at 3 weeks and in 40% of the B224-treated colonies at 5 weeks (Fig. 5A). These observations confirm the acquisition of the novel symbiont, but also suggest that it was lost over time. Because *Symbiodinium* B224 was not detected in any of the B224-treated colonies that were returned to the field and sampled after 14 and 28 wks (Fig. 5A), the novel symbionts that were experimentally acquired were lost over time. Although not detected with the cp-23S-rDNA primers, the novel D206 symbiont was detected in 80% of the colonies from the D206 treatment after 3 weeks of exposure using D-specific qPCR primers and in 40% of the colonies after 5 weeks (Fig. 5B). The colonies with D206 could not be returned to the field as this symbiont was isolated from a Pacific host. qPCR-specific primers were not developed for other symbiont types (A188 and B211), so it is possible that these strains were also acquired at similarly low levels.

Although one interpretation of the initial detection of symbionts was due to their presence in the coral gut or on the coral surface, this is unlikely as colonies were extensively rinsed in filtered seawater prior to sampling. Further, the novel symbionts were not detected in other treatments even though *P. divaricata* was exposed to the same concentration of all novel symbionts (1000 cells ml^−1^). Additionally, only B224 was detected using the less sensitive cp-23S-rDNA technique (relative to qPCR) and when examined with qPCR, symbionts were still detected after a 5 d period without exposure to these *Symbiodinium* which would have allowed the corals to clear their guts of any ingested *Symbiodinium*.

## Discussion

This study provides the first experimental evidence that although some scleractinian corals are capable of acquiring symbionts from the water column after a bleaching episode, this acquisition may be temporary. These findings have important implications about the response of corals to climate change. Firstly, these results establish that although scleractinian corals such as *Porites divaricata* are able to acquire novel *Symbiodinium* from the surrounding environment, the acquisition is transient, with the normal *Symbiodinium* assemblage being reestablished over weeks and months. The subsequent loss of the novel symbiont types over time may be due to an inability of the novel symbionts to multiply in the host or to compete with resident symbionts. Other instances where symbiont shifts have occurred have shown similar transitions back to the original symbiont community [10], [11], [28], [29]. This study, as others [11], [30], suggests that the acquisition of new symbionts does not provide a stable mechanism of acclimatizing to increasing SSTs. *P. divaricata* appears unlikely to rely on symbiont switching to ameliorate the effects of climate change on reefs. Corals may acquire symbionts from the environment, but these could be transient infections that are not maintained in a stable symbiosis and thus provide little hope of enhancing a coral's ability to acclimatize to predicted temperature increase associated with global warming.

Secondly, *Symbiodinium* differ in their physiological response to stressors [31], and members of *Symbiodinium* D, presumed to be a stress-tolerant clade, are reported to predominate in corals subjected to elevated temperatures and thermal bleaching [6], [7], [9], [10], [22], [32]. This has led to the hypothesis that *Symbiodinium* D may lessen the effects of climate change on reefs [6]. Since *P. divaricata* initially acquired *Symbiodinium* D, our results could be interpreted as supporting the hypothesis that *Symbiodinium* D may aid the coral host under conditions of thermal stress. However, *P. divaricata* did not readily acquire *Symbiodinium* D and its acquisition was transient and at low levels, only detectable with D-specific qPCR. An alternative hypothesis that our results do not reject is that Clade D is an opportunistic species that takes advantage of the heat stressed symbiosis (29), but further experimentation will be needed to accept or reject this hypothesis.

Finally, these results demonstrate that the corals did not acquire *Symbiodinium* indiscriminately. When colonies were exposed to a range of *Symbiodinium*, only those colonies in the B224 treatment acquired the novel symbionts as detected using cp-23S-rDNA screening, indicating that only B224 was taken up in large numbers. *Symbiodinium* B224 may have been acquired more readily because it is a Clade B symbiont, the clade that naturally predominated in this host coral, and B224 may be physiologically similar to the original symbionts (B170). Because we did not measure physiological parameters, we do not know if B224 provided the host with interim benefits. Yet, we observed selectivity even within Clade B as *Symbiodinium* B224 was not retained when corals were returned to the field, and B211was not acquired at all.

The findings presented here support the hypothesis that changes in the most abundant symbionts observed in post-bleaching recovery of adult scleractinians probably result from the survival and population growth of *in hospite* symbionts rather than the acquisition of novel types from the environment [7], [33], [34]. Multiple studies have demonstrated selectivity in symbiont acquisition during early ontogeny [14], [15], [35] and recent evidence indicates a genetic basis for this selection as reflected in differential gene expression in the presence of non-compatible symbionts [36], [37]. Variation in symbiont tolerances to heat stress among within-clade symbiont types (i.e. [11]) suggests that the host may acclimatize to environmental changes by shuffling symbiont composition toward closely related symbionts (intra-cladal types) where evolutionary divergences are not as great as between those symbionts of different clades. However, the ability to associate with novel symbionts would most likely have to evolve over time scales longer than the ecological times scales over which global warming is acting.

Although we have demonstrated that an adult scleractinian coral, *P. divaricata*, can acquire novel symbionts from the environment, it is noteworthy that these were not maintained through time. If similar interactions are encountered in other host species, it would suggest that corals will not be able to acclimatize or adapt to global warming by changing symbionts. If so, only coral species that already host heat-tolerant symbiont strains will acclimatize to the increasing temperature and other stresses predicted worldwide over the next 30–50 years, although even these may not be able to tolerate the temperature increases that are predicted [1]. Thus, these findings suggest that unless action is taken to curb global warming, the outcome of this will be a loss of coral reef biodiversity, leading to reefs that are very different from those that now exist.
